# Strontium-Substituted Calcium Orthophosphates: Structure, Stability, Morphology, and Biomedical Applications

**DOI:** 10.3390/ijms26125886

**Published:** 2025-06-19

**Authors:** Adriana Bigi, Elisa Boanini

**Affiliations:** Department of Chemistry “Giacomo Ciamician”, Alma Mater Studiorum—Università di Bologna, Via Gobetti 85, 40129 Bologna, Italy; adriana.bigi@unibo.it

**Keywords:** bone, Sr ion, coating, cement, scaffold, apatite, calcium phosphate

## Abstract

Strontium ions are of great interest because of their beneficial role in bone remodeling. This paper provides an overview of the present knowledge on the substitution of calcium with strontium in calcium orthophosphates. In particular, attention is focused on the influence of the substitution on the structure, morphology, and stability of calcium orthophosphates, as well as on the impact of strontium-substituted calcium phosphates on biomaterials in bone substitution/repair.

## 1. Introduction

Strontium is widespread as a trace element in a variety of rocks and soils, as well as water and groundwater [1]. It belongs to the same alkaline earth group as calcium and displays the same ionic charge (+2), and like calcium, it is considered a bone-seeking ion. Bone is a living tissue that modifies itself through a continuous remodeling process that controls its mass through the maintenance of a delicate balance between bone formation, which is operated by osteoblasts, and bone resorption due to osteoclasts. Strontium distribution in bone is related to tissue turnover; its concentration is greater in new bone than in old bone and greater in trabecular tissue than in compact tissue [2,3,4]. Relatively low doses of strontium promote bone formation, whereas relatively high doses have been reported to provoke impaired bone mineralization, such as that which occurs in osteomalacia [5]. The beneficial role of strontium in bone turnover occurs through two pathways: the promotion of bone formation thanks to improvement in osteoblast proliferation and differentiation and the inhibition of bone resorption through the enhancement of osteoclast apoptosis and the suppression of their differentiation [6]. Moreover, several studies have demonstrated strontium’s ability to promote angiogenesis [7].

In old age, bone remodeling becomes faster with a consequent loss of mass, reduced strength, and general degeneration of bone tissue, which leads to osteoporosis [8]. The results of a number of studies that indicated a beneficial role of strontium in promoting the proper balance between bone formation and resorption [9,10] promoted the development of a drug, Strontium Ranelate, which was widely utilized for the treatment of post-menopausal osteoporosis for several years [11]. However, this drug presented significant adverse effects, and its use for treatment was stopped in 2014 [12]. This has led researchers to consider the local delivery of strontium through functionalized biomaterials as a possible alternative treatment for bone diseases that are characterized by abnormal mass loss [13,14,15].

A vast portion of biomaterials that are used for the substitution/repair of the hard tissues of vertebrates is constituted of calcium orthophosphates (CaPs), which display very good biocompatibility and bioactivity. The inorganic phase of bone, as well as that of enamel and dentine, is indeed made up of a calcium phosphate similar to synthetic hydroxyapatite: Ca_10_(PO_4_)_6_(OH)_2_ (HA). Biological apatites present several differences in comparison to synthetic ones, such as a lower crystallinity and no stoichiometry [16,17]. Most of the investigations into CaPs have been performed on HA, but the interest in this class of compounds also involves non-apatitic CaPs, which are not usually present in normal calcified tissues and only occur in pathological mineralization [18]. Non-apatitic CaPs (Table 1) generally exhibit greater solubility and lower stability than HA; furthermore, most of them transform into more stable phases under physiological conditions [19]. The functionalization of CaPs allows us to couple their good biological performance with the peculiar properties of the functionalizing agent [20]. In particular, in vitro and in vivo studies have demonstrated that strontium’s association with CaPs enhances bone formation and inhibits bone resorption [12,15].

The functionalization of CaPs with strontium can be performed through ionic substitution or doping. Ionic substitution occurs when a foreign ion (Sr^2+^) enters into the structure of a crystalline compound, replacing an ion (Ca^2+^) in its regular position. On the other hand, “doping” is generally used to indicate a simple adsorption of the functionalizing ion on the surface of the crystals [16]. Ionic substitution modifies some of the properties of the compound, but the resulting crystalline phase is isomorphous with that of the not-substituted compound.

The present work is aimed at analyzing the structural, morphological, and biological modifications induced by the substitution of calcium with strontium in CaPs; therefore, it will not take into consideration studies on doped materials or those in which ionic substitution is not suitably supported by experimental data. In fact, doped materials allow for poor control of the position and interaction of the metal with respect to the crystalline structure, which could lead to poor reproducibility in the synthesis of the material and, consequently, to significant variability in its chemical, physical, and biological performance. Similarly, co-substitution studies do not allow us to identify the exact position of the different metals within the calcium phosphate structure since it is very hard to discriminate between the substituent ions based on the crystallographic data, leading to poor accuracy in the description of the material. Furthermore, co-substitution with ions that have different charges or ionic radii often causes a general decrease in the crystallinity of the material, which could be beneficial for the cellular response but still creates problems related to control in the synthesis of the material and, consequently, in its reproducibility.

Therefore, this paper is focused on the substitution of calcium with strontium in CaPs, as well as the influence of the substitution on their structure, morphology, and stability. Furthermore, the last section will describe the applications of Sr-substituted CaPs in the field of biomaterials for bone tissue replacement/repair.

## 2. Structure and Stability

The components of CaPs differ in several aspects, including structure, stability, and morphology. The identification of the different CaPs is based on their characteristic X-ray powder diffraction patterns, since they present with different structures. HA can crystallize in the monoclinic form in space group P2_1_/b, as well as in the significantly more stable hexagonal form in space group P6_3_/m; OCP displays a triclinic structure in space group P-1; the β form of TCP is rhombohedral in space group R3c; the α form is monoclinic in space group P2_1_/a; DCDP and its anhydrous form, DCPA, crystallize in the monoclinic Ia group and the triclinic P-1 space group, respectively; the crystals of MCPM, as well as those of its anhydrous form, MCPA, belong to the triclinic P-1 space group; and, finally, TTCP crystallizes in the monoclinic P2_1_ space group [21].

The number of crystallographic independent cation sites varies from 1 in DCPD and MCPM, 2 in DCPA, MCPA, and HA, 5 in β-TCP, 8 in OCP and TTCP, to 18 in α-TCP [21]. Also, the coordination number of the independent cation sites varies; although the most frequent coordination numbers are six and seven, smaller and larger numbers are present, such as five in α-TCP and nine in β-TCP [21].

The stability of these compounds in aqueous solution follows the order HA > OCP > TTCP > β-TCP > α-TCP > DCPD > DCPA > MCPM = MCPA [22], spanning from the lowest solubility of HA (pKps = 116.8) to the highest solubility of MCPM (pKps = 1.14). The two crystallographic forms of TCP, as well as those of TTCP and MCPA, can only be obtained through the solid-state reaction or heat treatment of other phases. The pH of stability of the phases that can be synthesized in solution decreases from that of HA (9.5–12), OCP (5.5–7), and DCPD (2–6), to that of MCPM (0–2) [22].

Moreover, most of the less stable phases undergo phase transition to more stable phases in solution [23,24,25,26]. The final phase(s) of the hydrolysis reaction of α-TCP, OCP, DCPD, and TTCP in aqueous solution depends on the pH, temperature, and presence of foreign agents in the solution [24,26,27,28,29].

Most of the literature on ionic substitutions into CaPs concerns hydroxyapatite, which is not surprising since it is the most similar to the inorganic phase of bone; moreover, the flexibility of its structure allows it to host a variety of substituents [16,30,31].

**Figure 1:**
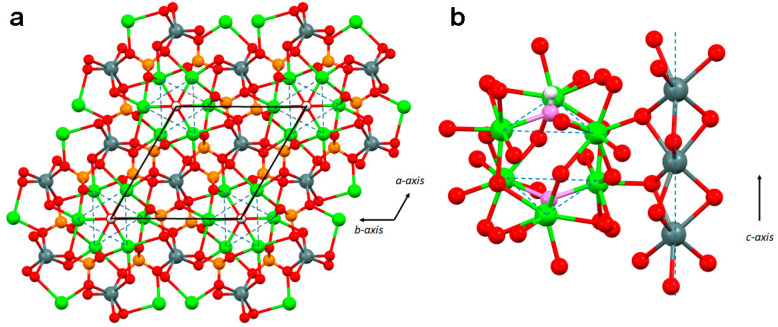
HA crystal structure: (**a**) view along the *c*-axis and (**b**) in an orthogonal direction. Grey: Ca(I); green: Ca(II); red: O; orange: P; white: H. In (**b**), the color of the oxygen atoms of OH^−^ is lilac. The solid lines in (**a**) mark the projection of the unit cell on the (001) plane, whereas the dotted lines form triangles that connect Ca(II) atoms lying on the same plane.

Sr_10_(PO_4_)_6_(OH)_2_ is isomorphous with Ca_10_(PO_4_)_6_(OH)_2_, where strontium can replace calcium in the whole range of the composition. Substitution causes a linear expansion of the lattice constants, which is more in agreement with the larger ionic radius of Sr (0.118 nm) than that of Ca (0.100 nm) [32,33,34,35]. Most of the studies on the structural modifications induced by Sr on HA indicate that at relatively high concentrations, it prefers to occupy site M(2), whereas at low concentrations (less than 5 at%), it preferentially occupies site M(1) [32,33,34,35,36,37,38] (Figure 1).

On the other hand, the results of the principle calculations performed by Matsunaga and Murata [39] showed that M(1) displayed the lowest energy state at any Sr concentration. Again, Wang et al. [40], on the basis of the results of an experimental, theoretical, and simulative study, suggested that Sr preferentially enters the M(1) site with a Sr content of 10 at%, thereby causing an anisotropic expansion of the unit cell. Furthermore, a decrease in the coherent length of the perfect crystalline domains was observed at relatively low concentrations [32]. It was suggested that the variation in the preferential site occupation of Sr ions in the HA structure is related to the different characteristics of the two metal sites [32]. The four M(1) sites are aligned in columns parallel to the crystallographic *c*-axis in a less flexible arrangement than that of the six M(2) sites, which are located at the apexes of “staggered” equilateral triangle centered on the OH channel (Figure 1). However, the M(1)-O distances are longer than the M(2)-O distances and can host the large Sr ion until its content provokes repulsions between the atoms at the M(1) site, after which it is better accommodated at the M(2) site.

The increasing replacement of calcium with strontium ions was observed to cause a significant increase in the solubility of the substituted HA, which was ascribed to a destabilization of the structure due to the presence of the bigger substituent ion [41]. The results of infrared and Raman absorption studies confirmed the expansion of the unit cell and the structural distortion induced by strontium [32,42]. In particular, the shift of the phosphate absorption band to lower energies was suggested to be due to a decrease in the repulsion between anions, which are more separated when the structure hosts the large strontium ion [32].

The results of the principle calculations performed by Matsunaga and Murata [39] suggest that the substitution of calcium with strontium could be easier in the OCP structure than in the HA structure. In fact, the substitution energies of the M(3), M(4), and M(8) sites of OCP are lower than those of the M(1) and M(2) sites of HA, whereas the substitution energies of the other cation sites of OCP are similar to those of HA. However, the results of the experimental investigations indicate that the substitution in OCP only occurs in a limited range; the upper limit has been reported to amount to 5, 7.4, or up to 23.25 at%, depending on the synthesis route [43,44,45,46]. However, the degree of crystallinity decreases significantly for concentrations in solution greater than 15 at% [46]. Strontium substitution induces an expansion of the unit cell of OCP that is in agreement with its greater ionic radius.

**Figure 2:**
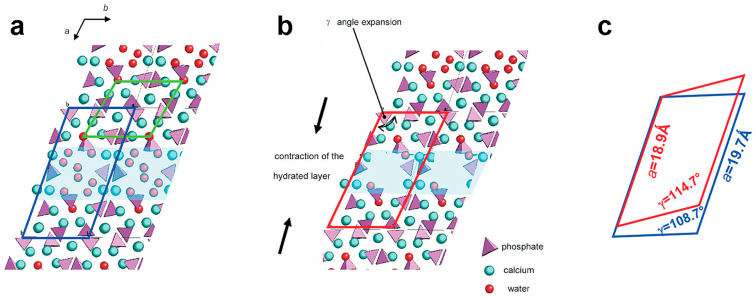
Possible modifications of OCP structure leading to the formation of “collapsed OCP”: projection of the structures of (**a**) OCP and (**b**) “collapsed” OCP along the *c*-axis: the green line in (**a**) indicates the unit cell of HA in the apatitic layer; (**c**) comparison between the unit cell of OCP (blue line) and that of “collapsed OCP” (red line). Reprinted (adapted) with permission from reference [44]. Copyright 2010 American Chemical Society.

The Rietveld refinement of OCP with a strontium content up to 10 at% indicates that strontium enters the M(3), M(4), and M(8) sites, as well as the M(7) site [47], which has a relatively high substitution energy [39]. Shi et al. [48] reported that OCP synthesized in the presence of 5 at% of strontium in solution showed an increased thermal stability in comparison with pure OCP on the basis of the modest shift to the higher temperatures of the thermal processes, which are associated with the removal of water molecules and lead to the conversion to poor crystalline hydroxyapatite. On the other hand, the TG-DTG plot of OCP synthesized in the presence of 15 at% of strontium in solution by Boanini et al. [44] shows a clear shift in the thermal processes to lower temperatures in comparison to that of pure OCP, indicating reduced stability. Furthermore, the structural analysis performed by the same authors [44] during heat treatment up to 300 °C confirmed the decrease in thermal stability provoked by the strontium substitution and allowed the authors to identify a new crystalline phase, “collapsed OCP”, which occurs during the thermal transition to apatite. During the structural transition to “collapsed OCP”, the unit cell of OCP, where “apatitic layers” alternate with “hydrated layers”, undergoes a modest reduction, as well as a different inclination (Figure 2).

**Figure 3:**
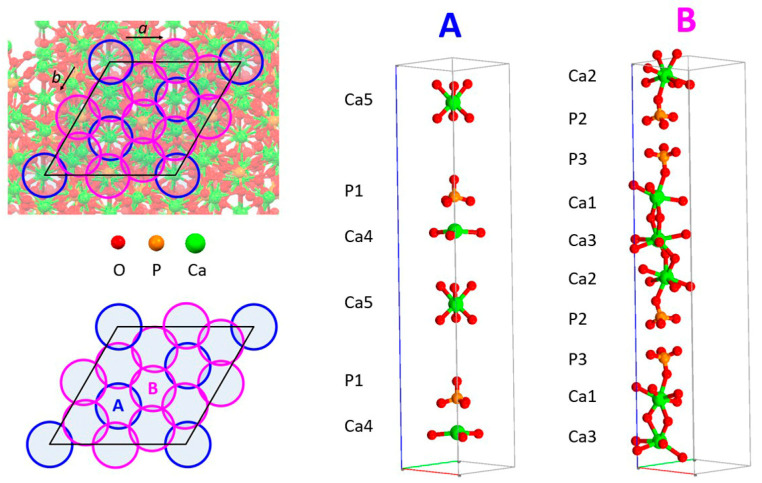
(**Left**): Projections of the β-TCP unit cell along the *c*-axis. (**Right**): View of the characteristic atoms in the A- and B-type columns. Reprinted from reference [55] and licensed under CC-BY 4.0.

Strontium can substitute calcium both in the β and in the α form of TCP, although in significantly different amounts. The structure of β-TCP hosts five crystallographically independent cation sites and three different phosphorous sites. While M(1), M(2), and M(3) occupy general positions and are coordinated to eight/nine oxygens, M(4) and M(5) are in special positions. The coordinations of M(5) and M(4) are approximately six and nine, respectively. Furthermore, only half of the M(4) site is occupied [49]. The substitution of calcium with strontium in β-TCP has been obtained through synthesis in solution, followed by heat treatment at high temperatures, as well as through solid-state reactions at high temperatures and by using a mechano-chemical activation method. The results of the different studies generally agree on the possibility of strontium ions entering this structure in relatively large amounts, up to about 80 at%, provoking a linear expansion of the cell parameters [50,51,52,53,54,55,56]. Structural refinements indicate a clear preference for strontium at the M(4) site. In particular, Renaudin et al. [51] analyzed whitlockite that contained about 4.4 at% Sr and was obtained by heat treating Sr-HA synthesized through a sol-gel reaction, which also yielded secondary phases, namely, strontium-substituted HA together with an amorphous phase. They concluded that most Sr (75%) is in column A (which contains M(4), M(5), and P(1)) (Figure 3), with the M(4) site containing 25% Ca and 25% Sr, which improves the geometrical shape of the cation site and, as a consequence, stabilizes the whitlockite structure. In *β*-Ca_2_Sr(PO_4_)_2_, synthesized via a solid-state reaction, M(4) was found to be occupied only by strontium, whereas M(5) only contained calcium [52]. Moreover, the sample with a higher strontium content, about 77 at%, was better refined using a structure that is topologically similar to β-TCP but contains a symmetry center and a unit cell characterized by a half c-parameter and volume (β’-TCP, space group *R*-3*m*) [52].

**Figure 4:**
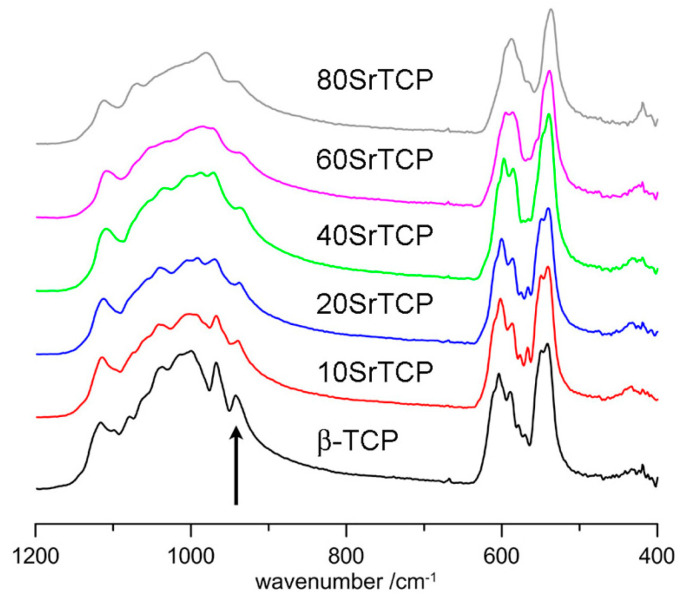
ATR-FTIR spectra of β-TCP at different Sr contents. The arrow indicates the ν1 band at 943 cm^−1^ in the spectrum of β-TCP. Reprinted from reference [55] and licensed under CC-BY 4.0.

A more recent study confirmed and detailed these results [55]. In particular, the use of the β’-TCP structure provided better fitting for a sample containing 80 at% Sr, although it could not be utilized for the refinement of samples at lower Sr contents, whose reflections are better indexed using the noncentrosymmetric β-TCP structure. The results of the powder fitting refinements show that at low concentrations, strontium displays a clear preference for M(4) (A column); after increasing the degree of substitution, it goes to M(3) (B column), and at even greater contents, it also fills M(1) and M(2) (B column). The absence of Sr in M(5) is consistent with the short M(5)-O distances, which are not suitable for the large Sr ion. The infrared absorption spectra of β-TCP at increasing strontium contents display a broadening of the absorption bands and a shift to lower wavenumbers. In particular, the shift of the 943 cm^−1^ band to 936 cm^−1^ due to P(1) is in agreement with the preferential strontium occupancy of the Ca(4) site (Figure 4) [55].

Pure β-TCP is stable up to 1125 °C, and above this temperature, it converts into the α form [21]. The value of the transition temperatures varies in the presence of impurity. In the case of strontium, Somers et al. [57] reported a slight increase in this temperature for strontium substitution of 4.5 at%, as well as a more significant increase when the foreign ion content reaches 9 at%, suggesting a stabilizing effect of Sr on the structure of β-TCP, which is in agreement with the report by Renaudin et al. [51].

**Figure 5:**
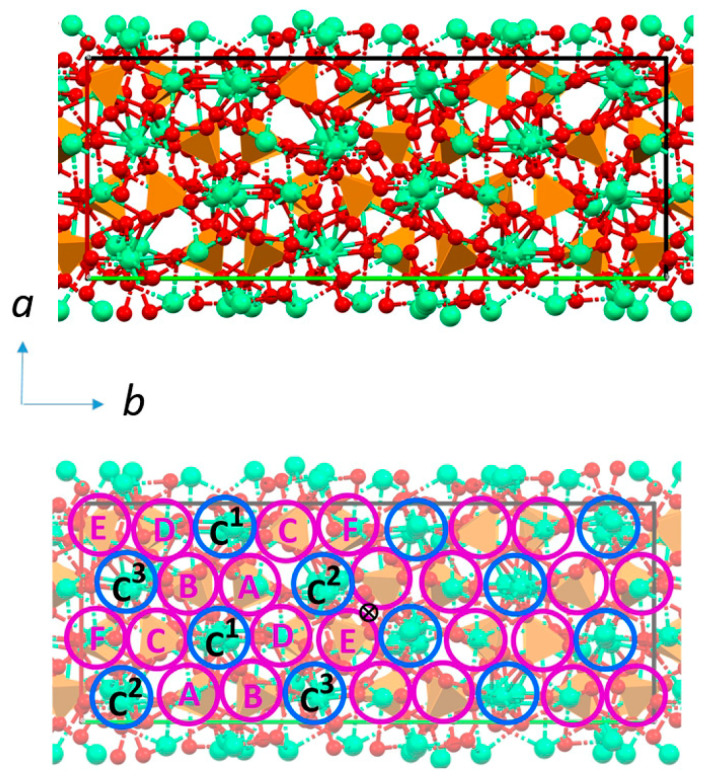
Projection of the *α*-TCP structure along the *c*-axis. The cation–cation columns (blue circles) are surrounded by cation–anion columns (magenta circles). Cation sites in cation–cation columns: C1: Ca8, 9, 10, 11; C2: Ca2, 3, 4, 5; C3: Ca14, 15, 16, 17. Calcium and phosphate ions in cation–anion columns: A: Ca7, P3, P4; B: Ca12, P5, P6; C: Ca6, P1, P2; D: Ca13, P7, P8; E: Ca18, P9, P10; F: Ca1, P11, P12. The cross is the center of symmetry. Plots were created with Mercury software (CCDC, version 2022.3.0). Reprinted from reference [59] and licensed under CC-BY 4.0.

The amount of strontium that can substitute calcium in the structure of α-TCP is significantly smaller than that hosted by β-TCP. The results of studies performed on samples synthesized using different methods show that Sr can enter into the structure of α-TCP at up to 10 at% [58,59]. The substitution provokes an increase in the unit cells and different shifts in the powder XRD reflections, resulting in different variations in the lattice constants: the *a*-parameter undergoes a modest reduction, whereas the *c*-parameter, and even moreso the *b*-parameter, increase with increasing Sr content [59]. In agreement with this finding, Rietveld refinements indicate that substitution occurs preferentially at three cation sites, namely, M(17), M(11), and M(5), which Matsunaga et al. [60] indicated to be among those that are energetically favorable. The 18 cationic sites of α-TCP are distributed in three different cation–cation columns, surrounded by six cation–anion columns, as shown in Figure 5. M(17), M(11), and M(5) are each located in a different cation–cation column and are characterized by relatively small bond valence sums, long mean Ca–O distances, and wide Ca–O distance distributions [59]. Isothermal calorimetry curves indicate that strontium slows down the rate of dissolution of α-TCP and the successive precipitation to calcium-deficient HA [58]. Moreover, strontium was found to slow down the hydrolysis reaction of α-TCP both in mildly acidic solution and in physiological solution [59].

Both brushite and monetite structures can host strontium ions, although in different contents: the upper limits of Ca replacement with Sr were reported to amount to about 38 at% in DCPD and 100 at% in DCPA. Because of the wide employment of DCPD and DCPA in the composition of calcium phosphate bone cements [61,62], strontium doping has also been explored in these compounds, although only a few studies have provided a structural characterization suitable to state whether the foreign ion is regularly incorporated into the CaPs structures. Sayahi et al. [63] synthesized brushite in the presence of increasing amounts of Sr ions in solution and obtained a single DCPD phase with slightly enlarged cell parameters only for Sr contents up to 4.7 at%, whereas greater concentrations provoked the precipitation of secondary phases. The presence of secondary phases, in particular that of strontium hydrogenphosphate, was confirmed by the variation in the infrared absorption spectrum of DCPD in the 800–1300 cm^−1^ region [63].

At variance, a further study showed that strontium can substitute calcium in DCPD up to about 38 at% [64]. Rietveld refinements indicated an increase in the dimensions of the unit cell with increasing strontium substitution, which also provoked a shift in the (ATR)-FTIR absorption bands. In particular, the OH stretching at 3541 cm^−1^ shifted to higher wavenumbers, whereas the PO stretching at 986 cm^−1^ and the PO bending at 662 and 576 cm^−1^ shifted at lower wavenumbers due to a decrease in the anion–anion repulsion [64,65].

**Figure 6:**
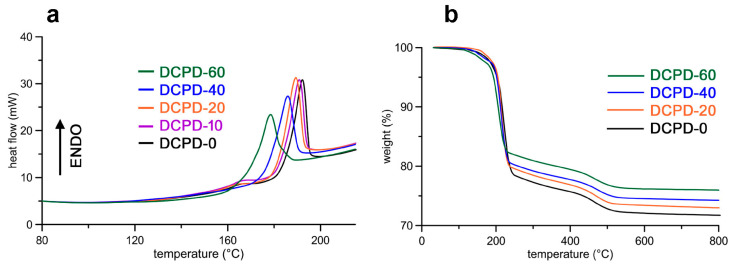
(**a**) DSC and (**b**) TG curves of DCPD samples synthesized in the presence of increasing Sr content. Reprinted with permission from reference [66]; Copyright 2021 from Elsevier Ltd. and Techna Group S.r.l.

At relatively low concentrations, strontium does not affect the hydrolysis reaction of DCPD in physiological solution, which leads to its transformation into OCP and HA. However, Sr-substituted DCPD with a Sr content of about 38 at% hydrolyzes to an unknown phase at 37 °C, whereas at 60 °C, it converts into Sr-substituted HA and Sr-substituted DCPA [64]. Moreover, the thermal stability of brushite decreases as a function of strontium content: the results of differential scanning calorimetry and thermogravimetric analyses clearly show that the temperature needed for DCPD to lose its two structural water molecules and convert into DCPA decreases with increasing Sr content (Figure 6) [66].

Strontium-substituted DCPD was successfully utilized to synthesize strontium-substituted DCPA through heat treatment [66]; however, the range of substitution into the monetite structure was limited by that of the starting DCPD. At variance, the direct synthesis of DCPA in solution in the presence of increasing strontium concentrations allowed researchers to verify that this ion can act as a substitute for calcium throughout the whole range of composition, thereby causing a linear expansion of the lattice parameters [66]. As previously observed for Sr-substituted HA [32], the mean dimensions of the coherent length of the perfect crystalline domains decrease with Sr content up to about 50 at% and then increase up to 100 at%. The structure of DCPA consists of an assembly of CaHPO_4_ chains, where calcium atoms occupy two crystallographically independent cation sites, M(1) and M(2), with similar mean Ca-O distances [67]. The results of full-pattern refinements do not indicate a clear preference of strontium for the seven-coordinated (M(1)) site or the eight-coordinated (M(2)) site when the Sr content is less than 50 at%, whereas site M(2) is preferred at relatively high Sr contents [66].

Due to their high solubility, MCPM and MCPA have limited practical interest, although they are used as components of bone cement. The use of TTCP in this field is even more widespread, but its ability to host foreign ions has rarely been investigated until now; in particular, Jayasree et al. prepared Sr-substituted TTCP with a strontium content up to 10 at% and reported that the substitution provoked an enlargement of the cell parameters [68].

## 3. Morphology

HA precipitated in aqueous solution under mild conditions consists of tiny crystals with a plate-shaped morphology that is elongated in a direction parallel to the crystallographic *c*-axis [69]. When the temperature is relatively low (room or physiological temperature), the poor crystallinity and stoichiometry cause the nanoparticles to have an irregular shape, which also occurs in biological apatites. In contrast, when the temperature increases to close to the boiling point, the as-prepared HA is stoichiometric, and the crystals show increasingly defined faces, edges, and vertices, reflecting the increased crystallinity. The final mean dimensions increase up to about 200 × 40 nm [32,70].

**Figure 7:**
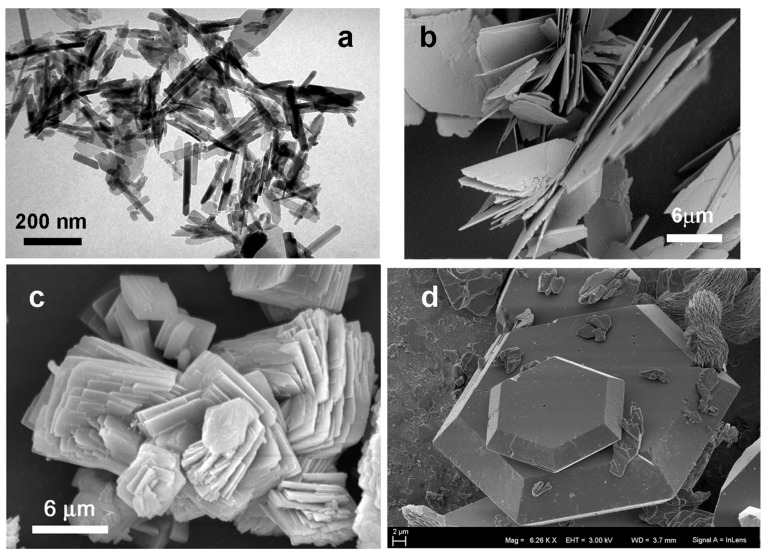
(**a**) TEM micrograph of Sr-substituted HA (Sr: 70 at%). Reprinted with permission from reference [32]; Copyright 2006 from Elsevier B.V. (**b**) SEM image of Sr-substituted DCPD (Sr: 38 at%). Reprinted from reference [64] and licensed under CC-BY 4.0. (**c**) SEM image of Sr-substituted DCPA (Sr: 38 at%). Reprinted with permission from reference [66]; Copyright 2021 from Elsevier Ltd. and Techna Group S.r.l. (**d**) SEM image of the nucleation of a crystal of HA synthesized in the presence of 53 at% Sr in solution. Reprinted from reference [85] and licensed under CC-BY-NC.

The insertion of strontium into the nanocrystal structures has different effects on morphology, depending on the amount of the substituting ions. At low Sr contents (0 < Sr ≤ 50 at%), the crystals display perturbed shapes and ill-defined edges compared with stoichiometric Ca_10_(PO_4_)_6_(OH)_2_, which is in agreement with the lower degree of crystallinity of the material. At relatively higher Sr contents (Sr ≥ 70 at%), the crystal dimensions increase with increasing Sr content, resulting in Sr-HA crystals that are larger than HA crystals and display mean dimensions of about 500 × 100 nm, as well as a very well-defined elongated shape (Figure 7a) [32,70]. It may be inferred that the morphological properties of Sr-HA crystals somehow reflect the increased degree of structural crystallinity of the totally substituted hydroxyapatitic phase.

The morphology of hydroxyapatite crystals has been widely explored in the literature. In many studies, however, the proposed synthesis methodologies have involved subsequent treatments or the use of specific additives in solutions, with the consequent modification of the morphology of the crystals, along with other properties of interest. In general, the sintering process, or whatever treatment is applied to the crystals at high temperatures, causes a significant increase in size, as well as a reduction in the specific surface area (from tens of m^2^/g to less than 1 m^2^/g) and the formation of rounded shapes when the faces of the pristine crystals are lost [71,72]. This occurs due to the extreme temperature involved in the melting and recrystallization of the solid. This physical phenomenon and its consequences seem not to be influenced by the presence of strontium in the structure of hydroxyapatite crystals; therefore, the Sr-apatite crystals display similar changes [71]. Elongated particle morphologies (wires, ribbons, and tubes) have been obtained using organic modifier-assisted HA synthesis methods, sometimes in combination with solvothermal treatment and the microwave-assisted method [73,74,75,76], in which the role of strontium has not been explored yet. Recently, Ye et al. developed a strategy to mineralize collagen with Sr-doped hydroxyapatite using the polymer-induced liquid precursor (PILP) process. Interestingly, the increase in the Sr substitution in crystals gradually inhibited the mineralization degree, changed the crystal size and crystallinity, and altered the nanocrystal orientation relative to the collagen fibers [77].

Contrary to the ionic substitution in the hydroxyapatite structure, which can reach completeness, the substitution in the OCP structure is very limited and, in general, causes a progressive and rapid decrease in crystallinity due to geometric and steric reasons. Depending on the experimental conditions, OCP precipitates from direct synthesis as individual micrometric platelets, which are elongated along the crystallographic *c*-axis direction, and can eventually be assembled into spherules with diameters up to hundreds of microns and made up of long blades that originate from a common center [78]. The morphological modifications provoked by Sr insertion into the structure proceed in parallel with the decrease in crystallinity, so that the substituted crystals show reduced dimensions and damaged terminated ends [44]. The inclusion of carbamide during the synthesis procedure maintains the plate-like crystal shape with a laminar structure; however, as the Sr content in the structure increases, the width of the crystals increases and the length decreases, thus leading to lower length/diameter ratios. Again, at relatively high Sr contents, the crystals appear smaller, with irregular edges and imperfect morphologies [46]. These results show that excessive Sr content would compromise the integrity of OCP crystals, which is consistent with the XRD results. Teterina et al. synthesized Sr-substituted OCP from DCPD powder using a low-temperature chemical transformation method in sodium acetate solution in the presence of Sr^2+^ ions. It was observed that at low Sr contents (≤10 at%), the overall shape of the crystals was similar in its macrostructure to that of the DCPD, from which the material was synthesized, and the surface had a complex shape, which indicated inhomogeneity. With an increase in the strontium content up to 50 at%, the morphology became uneven, and the crystals had wide lamellar shapes and rough edges [45].

α and β-TCP morphological features are very different from those of other calcium phosphates and consist of nearly spherical particles, which are necked to aggregates of irregular shapes. The dimensions of the particles can be slightly submicrometric or micrometric depending on the synthesis procedure [55,79,80]. The morphology of β-TCP does not seem to be significantly affected by the presence of a low amount of Sr, whereas the products synthesized at increasing Sr contents appear to be constituted of more dense blocks due to the greater tendency of Sr-substituted β-TCP powders to agglomerate. However, the characteristic round-shaped morphology is always retained [53,55,56]. Similarly, the substitution of Ca^2+^ with Sr^2+^ did not change the morphology of α-TCP. It has been reported that the average particle size is not affected up to about 5 mol% Sr substitution [81]; detailed morphological studies at higher Sr contents are lacking, although it was reported that Sr can enter into the α-TCP structure up to 10 at% [59]. This could be due to the fact that the main interest in this crystalline phase is its application in the production of bone cements, in which a higher amount of Sr might not be useful, as it could affect the mechanical properties and setting times.

Pure DCPD shows a characteristic plate-like morphology in which the crystals are very thin (less than 200 nm) and exhibit a wide face (tens of micrometers long in the orthogonal directions) that corresponds to the (010) crystallographic plane. As a result, the crystals have a strong tendency to form stacked superstructures, and their surfaces appear shiny. The substitution of calcium with strontium induces the aggregation of crystals that exhibit indented edges. The aggregation of crystals increases, whereas their dimensions decrease when the Sr content is increased, yielding spherulitic aggregates of relatively small crystals at Sr substitutions higher than 30 at% (Figure 7b). The aggregation of crystals was also previously observed in the presence of different additives, suggesting that the perturbation of the DCPD structure reduced the crystal growth and promoted the interaction of crystals [63,64].

When strontium-substituted DCPD is utilized to synthesize strontium-substituted DCPA [66], not only is the range of substitution into the monetite structure limited by that of DCPD but the morphology also resembles the plate-like crystals of the corresponding DCPD samples before heat treatment. In particular, DCPA samples with increasing strontium contents confirm that the foreign ion promotes the aggregation and layering of the crystals [66].

At variance, the morphology of DCPA crystals obtained via direct synthesis spans from nanorods to fiber-like to rhombohedral/parallelepipeds [66,82,83]. Sr substitution has been explored in synthesis conditions that yield rhombohedral/parallelepipeds crystals of pure monetite [66]. In this case, the crystals exhibit a layered morphology, with a width of about 5–10 μm and a thickness of the layers, which exhibit a smooth surface, of 0.5–1 μm. The presence of strontium seems to promote the trend of the crystals to exfoliate; the layers become increasingly numerous and thin with increasing Sr content (Figure 7c). Simultaneously, crystal fragmentation increases, and rod-like crystals detach from the layers. This experimental setup allows for a complete Sr substitution to Ca, which corresponds to rod-like crystals with mean dimensions of 5 × 0.5 μm. Adawy and Diaz prepared Sr-containing monetite crystals with a two-stage procedure that consists of a reaction between β-tricalcium phosphate and monocalcium phosphate monohydrate in a mortar, followed by a hydrothermal autoclave reaction in the presence of Sr(NO_3_)_2_. Cryo-HRTEM observations showed a layered plate-like structure of the synthesized crystals, and selected area electron diffraction (SAED) resulted in polycrystalline diffraction patterns that could certainly be attributed to monetite. These patterns also displayed a little expansion in the d-spacing values, indicating the presence of Sr in the lattice [84].

The crystal growth of calcium phosphates is also of great interest in biomineralization and is important for understanding the physical mechanisms that form the basis of crystal growth and dissolution, as well as phase stability. A recent study used synthetic calcium phosphate gels and substitutional strontium ions to tailor crystal growth with a biomimetic approach [85]. Strontium presence significantly influences the morphological properties of calcium phosphate crystals, leading to the formation of highly symmetric structures with pseudo-hexagonal or hexagonal shapes (Figure 7d).

In general, apart from the numerous studies regarding substituted strontium apatite, few works are present in the literature regarding careful morphological studies on the other calcium phosphates, although they are also widely used in association with strontium for various biomedical applications.

## 4. Applications for Biomaterials

Due to their peculiar properties, a wide variety of studies have been conducted to investigate the applications of CaPs in the field of biomaterials for hard tissue substitution/repair. Moreover, there has been great interest in exploring the possibility of improving their performance through their functionalization with additives, such as polymers, proteins, polysaccharides, as well as biologically relevant ions and metallic particles [20,86,87,88,89]. In this field, an important part of research activity concerns functionalization with Sr ions. In particular, the substitution of Ca with Sr in CaPs has been investigated in calcium phosphate bone cements (CPCs), scaffolds, and coatings.

### 4.1. Cements

CPCs are proposed for implant fixation as an alternative to the most utilized polymethylmethacrylate (PMMA). Indeed, CPCs have the advantage of undergoing a non-exothermic reaction at variance with PMMA and are biocompatible and bioactive. They are based on the reaction between one or more CaPs and a liquid phase, yielding a paste that stiffens with time to produce a solid calcium phosphate. The proposed compositions of cement powders include α-TCP, TTCP + DCPA, TTCP + DCPD, and β-TCP + MCPM; the liquid phase can be made up of just water, as well as an aqueous solution [62,90]. Whatever the composition of the solid and the liquid phases, the possible final phases after their mixing, setting, and hardening are limited to brushite and apatite.

The influence of Sr ions on the main properties of CPCs, which include setting times, degradation, mechanical properties, injectability, and biological performance, has been tested by introducing them either in the solid phase or in the liquid phase [61]. However, a limited number of these studies discriminate between Sr substitution or doping. Moreover, while Sr substitution in the components of the solid phase can be easily determined, the final phase is often poorly crystalline and hinders a proper structural investigation. An important exception is given in the work by Gelinsky et al., who published a series of studies on a cement-obtained mixing a powder containing α-TCP, DCPA, CaCO_3_, and HA, with an aqueous Na_2_HPO_4_ solution [91,92,93,94]. Sr ions were introduced as SrCO_3_ in an amount that resulted in up to 2.21 at% of Sr in the cement. The solid phase obtained after hardening was HA with enlarged cell parameters, indicating a partial substitution of Sr for Ca [91]. Tadier et al. [95] proposed an unusual cement powder composition, namely, vaterite + brushite (1:1) mixed with distilled water, as the liquid phase; it was enriched with Sr, which was added as SrCO_3_ in the solid phase or as SrCl_2_ in the liquid phase, up to 8 wt%. The results indicated that SrCO_3_ did not participate in the setting reaction; at variance, Sr introduction into the liquid phase yielded HA as a single phase, with enlarged cell parameters. Strontium incorporation into the structure of HA up to 10 at% was also reported for a cement obtained from Sr-substituted TTCP mixed with a liquid phase containing Na_2_HPO_4_ and citric acid [68]. At variance, Guo et al. [96] did not evaluate cell parameters and observed only a shift in the 002 XRD reflection of HA in the cement obtained from TTCP + DCPA + SrHPO_4_ as starting powder.

A further CaP utilized as a component of CPCs is Sr-substituted β-TCP [97,98,99,100,101]. Most of the compositions used in these studies also involved the presence of MCPM in the solid phase and produced cements that contained DCPD as the main component. In particular, Sr substitution in the final DCPD phase was demonstrated by Alkhraisat [97,102]. DCPD characterized by a modest increase in the cell parameters in comparison to pure brushite was also reported by Pina et al. [103] as the final phase of a cement obtained using Sr-substituted α-TCP as cement powder.

The cementitious reaction between Sr-substituted DCPD and TTCP yielded an apatitic cement in which the presence of Sr (15 at%) provoked an enhancement of compressive strength and exhibited a synergistic effect on osteogenesis, osteoclastogenesis, and angiogenesis [104].

In general, the inclusion of strontium in the cement composition was reported to provoke the release of relevant doses of Sr ions [91,105], increased mechanical strength [91,96,100,101,104,105,106], and radiographic contrast [91,106], while lengthening setting times [100,105] and improving injectability [100,105,106].

**Figure 8:**
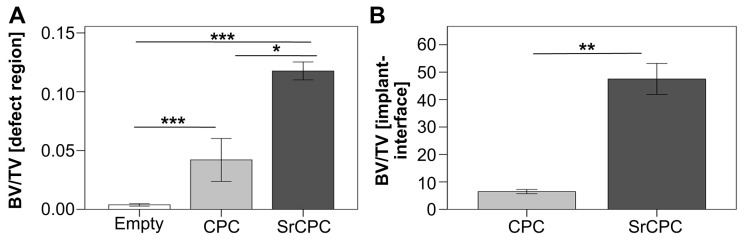
Histomorphometrical analysis of new bone formation of strontium-modified calcium phosphate cement (SrCPC) compared to a strontium-free calcium phosphate cement of otherwise similar composition (CPC) and an empty defect control group in the critical-sized metaphyseal defect in the femur of ovariectomized rats. (**A**): Initial fracture defect zone and (**B**): tissue–biomaterial interface. Asterisks indicate (*) *p* < 0.05, (**) *p* < 0.01, and (***) *p* < 0.001. Reprinted from reference [108] and licensed under CC-BY-NC-ND license.

The investigations into the influence of Sr presence on the biological performance of CPCs demonstrate that both the substitution and doping of this ion promote osteoblast proliferation and differentiation, as well as the formation of bones and blood vessels [12,15,107]. In particular, it was shown that the amount of new bone formation in a critical-sized metaphyseal defect in the femur of ovariectomized rats was significantly greater when the defect was filled with strontium-modified calcium phosphate cement (SrCPC) than when it was filled with calcium phosphate cement (CPC) (Figure 8) [108].

### 4.2. Scaffolds

A scaffold has been defined as “an artificial structure used to support three Dimensional (3D) tissue formation” [109]. The requirements of bone scaffolds include biocompatibility, osteoconductivity, osteoinductivity, suitable mechanical properties, porosity, and pore interconnection to allow for vascularization, as well as cell growth and migration [109,110]. In other words, an ideal bone scaffold should be as close as possible to bone tissue in composition, structure, morphology, and function [111]. The inorganic phase of bone is described using synthetic hydroxyapatite as a model, whereas the main component of the organic matrix is type I collagen. For this reason, scaffolds for the substitution/repair of the hard tissues of vertebrates quite often contain CaPs, either alone or in combination with natural and/or synthetic polymers. The methods of preparation of bone scaffolds are numerous and include gas foaming, solvent casting, freeze-drying, and 3D-printing [109]. In particular, 3D-printing can be achieved using a variety of different approaches: stereolithography, selective laser sintering, robocasting, inkjet bioprinting, microextrusion, and laser assisted bioprinting [110,112,113,114].

**Figure 9:**
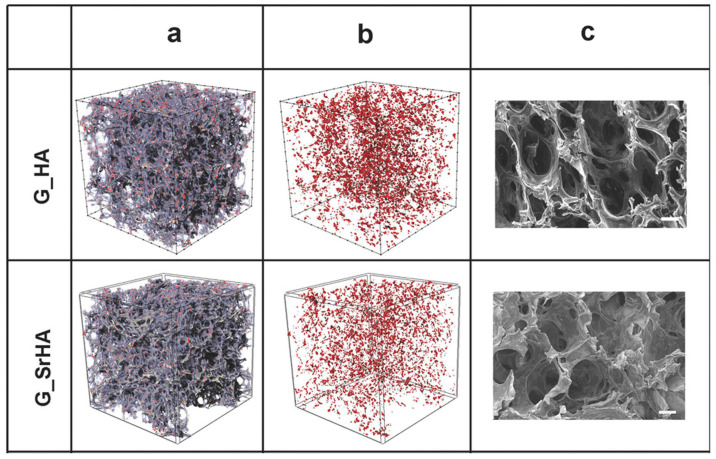
Three-dimensional μ-CT representation and SEM images of gelatin scaffolds reinforced with HA (G_HA) and Sr-substituted HA (G_SrHA). (**a**) Three-dimensional models of the scaffolds and (**b**) their inorganic phase (red) distribution; (**c**) SEM images. Scale bars = 200 μm. Reprinted with permission from reference [115]; Copyright 2018 from Wiley.

The freeze-drying of strontium-substituted HA with a Sr content of 10 at% mixed with gelatin was utilized by Panzavolta et al. [115] to prepare scaffolds with very high open porosities and an interconnectivity of 100% (Figure 9), which displayed a sustained Sr release in phosphate buffer solution. Analogously, Sr-substituted HA with varying Sr contents up to 100 at%, combined with chitosan and submitted to freeze drying, provided scaffolds with 3D-interconnected macropores with pore sizes of 100–400 nm [116]. Freeze-drying was also applied to obtain scaffolds that combined Sr-substituted HA (at two different Sr contents: 8 and 50 at%) with a blend of pullulan/dextran; the results of subcutaneous implantation in mice indicated that the presence of strontium stimulated the formation of osteoid tissue and vessels [117].

The co-precipitation of HA in the presence of Sr (up to 100 at%) during collagen fibril formation yielded collagen-inorganic phase composites, in which the mineral phase ranged from HA to poorly crystalline strontium-rich phases, up to a two-phase system, consisting of an amorphous strontium phosphate and crystalline Sr hydroxyapatite [118]. The composites exhibited an interconnected porosity and a sustained Sr release, which increased with the increase of the Sr content in the scaffolds. On the other hand, co-precipitation in a gelatin solution, followed by freeze-drying, was reported to produce a porous scaffold with an oriented microtubular structure, in which the precipitated HA displayed a shift in the powder X-ray diffraction reflections, indicating a partial substitution of Sr for Ca [119].

Sr substituted HA was combined with poly(trimethylene carbonate) (PTMC) and submitted to digital light processing (DLP) to obtain 3D-printed scaffolds, which demonstrated in vitro and in vivo osteoinductivity and osteoconductivity [120]. Significant osteogenic activity both in vitro and in vivo was also reported for scaffolds obtained through the motor-assisted manufacturing (MAM) of blends of poly(ɛ-caprolactone) (PCL) and Sr-substituted HA [121].

Sr-substituted HA with a Sr content of 20 at% was also employed to obtain a 3D biomimetic bone tumor model using an extrusion bioprinter and a RGD-enriched alginate-based ink [122]. This study demonstrated that the use of calcium phosphate nanocrystals and structural ion substitution is a promising approach to tune the behavior of 3D bioprinted constructs. In fact, the addition of NPs to the ink increased printing fidelity, in a particle type-dependent fashion and reduced swelling, regardless of particle size and shape. Furthermore, the addition of non-soluble biomimetic NPs allowed for the increased biomimicry of the models, favoring cell viability at all time points.

Recently, Sr-substituted calcium phosphates as components of bone scaffolds have also included Sr-substituted β-TCP and whitlockite [123,124]. In particular, the presence of Sr-substituted β-TCP in porous scaffolds that also contain methylcellulose (MC) and are prepared using a pneumatic fiber extrusion-based 3D printing device, was shown to modulate the porosity and microtopography of the scaffolds, which exhibited a sustained Sr release [123]. Sr-substituted whitlockite, with increasing lattice parameters on increasing Sr content up to 7.5%, were loaded in a porous cryogel composed of methacrylated gelatin (GelMA) and methacrylated chondroitin sulfate (CSMA). In vitro and in vivo results indicated that the promotion of osteogenic differentiation and the inhibition of osteoclast activity varied with Sr content, which produced the best results at a substitution of 7.5% [124].

It is worthwhile to cite the work of Zhao et al. [125], who fabricated porous whiskers of pure HA (WCP) and of Sr-substituted HA (SrWCP) using a hydrothermal treatment. The whiskers were implanted in an osteoporotic rat metaphyseal femoral bone defect model, and the results were compared with those obtained from a group that received WCP plus strontium ranelate drug administration. The SrWCP showed similar beneficial effects on the quality of osteoporotic bone as those of strontium ranelate treatment, along with minor adverse effects [125].

The vast number of papers on the effect of strontium on the biological performance of CaPs that contain bone scaffolds usually compare the results with those obtained in the absence of strontium. The comparison between the different influence of Sr substitution and Sr doping is not present in the literature. It would be an interesting topic, since, as reported above, Sr substitution modifies not only the structure but also the morphology and the solubility of CaPs and, as a consequence, Sr and Ca release.

### 4.3. Coatings

Nowadays, performing implant procedures, ranging from screw insertion to total joint replacement, has become almost “routine” in both orthopaedics and traumatology, as well as in dentistry. The success of reconstructive orthopedic surgery heavily relies on the mechanical and biological integration of the prosthesis with the host bone tissue, a challenge that is further complicated by the aging population and the increasing prevalence of conditions associated with altered bone metabolism. Calcium phosphate-based coatings have been shown to be able to promote osseointegration for the implants in cementless arthroplasty. Therefore, a large number of studies have been dedicated to this topic in the last two decades, especially those devoted to functionalization strategies with both organic molecules and metallic ions. Among the studied ions, Strontium has attracted significant attention because it exhibits some of the best properties for implant osseointegration in vivo [126].

The plasma spray coating process is one of the most commonly used in industrial applications that is able to yield a good bond between the substrate and coating with no microcracks at the interface. Therefore, it is commonly used for the production of hydroxyapatite coatings on metallic prostheses. Plasma-sprayed Sr-containing HA coatings (10 mol% Sr^2+^) or Sr and Zn or Ag co-doped HA were produced on titanium alloy substrates [127,128,129]. The coatings always exhibited good adhesion to the substrate and a rough surface, which is beneficial to their bonding with surrounding bone tissue in vivo. They were continuous and consisted of flat and smooth splats, as well as some spheroidized and partially melted fine particles, indicating that the powder had a high degree of melting during spraying. They displayed good mechanical properties and bioactivity in vitro. However, all the deposited layers showed poor crystallinity. When they were crystalline, secondary phases, such as alpha- and beta-tricalcium phosphate, were also found, and no evidence of Sr substitution in the structure of hydroxyapatite was provided [127,128,129]. Even when Mg alloy was plasma-sprayed with HA reinforced with Sr powder (4, 8, and 12 wt.%) to prevent its corrosion in a biological environment, no peak shift was observed in the XRD patterns [130].

An attempt to obtain novel Sr-TCP coatings on Ti6Al4V alloys utilizing Ionized Jet Deposition (IJD) technology showed a significant reduction in bacterial adhesion and biofilm formation for application in spine prostheses due to the presence of Sr. IJD is a plasma plume-assisted technology that is able to deposit the target on a substrate; however, experimental evidence showed the formation of an amorphous phase when it is applied to the deposition of calcium phosphates, no matter the starting crystalline phase of the target [131].

To the best of our knowledge, unfortunately, there are not any studies that demonstrate that plasma-assisted techniques can produce Sr substitution in calcium phosphate lattices. In contrast, several coating deposition techniques have been shown to be effective for producing strontium-substituted calcium phosphate layers.

Using biomimetic methods, it was possible to incorporate different amounts of Sr into the structure of the apatite layer by adding a soluble Sr-containing salt in the composition of the calcifying solution at a physiological temperature (37 °C). Despite the poor crystallinity of deposited HA, the XRD pattern showed a clear shift of the intense (002) reflection to lower 2θ angles due to ion substitution [132,133]. Also, the surface chemical analysis via XPS and TOF-SIMS provided information about the bonding state of Sr ions in the apatite structure and showed that the Sr is chemically bonded and successfully incorporated into the structure of the apatite [134]. The composition can be controlled by varying the concentration of strontium ions in the soaking medium, and the presence of Sr ions in the solution could inhibit the coating formation and result in a reduced thickness of the coating [132,133]. The immersion of Ti substrates in different precursor solutions at 70 °C produced a DCPD layer that turned to DCPA upon insertion of Sr ions into the calcifying solution [135,136]. The good crystallinity allowed for the application of the Rietveld refinement method to the XRD profiles for the cell parameter calculation of Sr-monetite, which resulted in significant expansion after Sr incorporation into the lattice. All Sr–monetite coatings showed a rombohedral/parallelepipeds crystal morphology, with smooth surfaces typical of monetite precipitation from solution [66,135]. Surprisingly, the thickness increased with the increase in Sr content, which was related to the crystal enlargement and high crystal alignment [136]. In vitro tests clearly showed a significant promotion of osteoblast differentiation and mineralization due to the presence of Sr in the composition of the biomimetic calcium phosphate coatings [133,135].

Also, the presence of Sr in the target of the co-sputtering technique allowed for the procurement of a clear ionic substitution in the final material, both using mixture targets of hydroxyapatite and strontium apatite [137] and hydroxyapatite and partially substituted hydroxyapatite [138]. All XRD peaks shifted to lower 2θ values with increasing Sr/(Sr + Ca) target ratios, which indicated Sr incorporation into the HA lattice. Despite the good crystallinity showed by XRD peaks, the SEM observation revealed that the surface was covered with globular particles. The different experimental conditions employed during deposition had a significant impact on the dynamics of the sputtering process and, as a consequence, on the Sr content, thereby yielding coatings with Sr/(Sr + Ca) ratios with almost the same or significantly lower values than the targets [137,138].

Pulsed electrodeposition was successfully used to synthesize strontium-substituted calcium phosphate coatings on titanium alloy substrates [139]. The Sr/(Ca + Sr) ratio of the coating was approximately equal to half of the Sr/(Ca + Sr) ratio of the solution, and strontium was homogeneously distributed in a poorly crystalline apatite phase that converted into two distinct crystalline phases (HA and β-TCP) after heat treatment. Although the cell parameters of HA were not evaluated, the change in the solubility of these coatings as a function of the chemical composition and, as a consequence, the amount and rate of strontium release in physiological media suggested Sr incorporation into the apatitic lattice [139]. Among the tested coatings, the 10% Sr coating exhibited the best properties for implant osseointegration in osteoporotic rats, confirming the highest activity of Sr in comparison to other metals [126]. Strontium-substituted calcium phosphate coatings with different structures were also prepared on the surface of the Mg matrix via a simple one-step electrodeposition method at different temperatures, which enhanced the poor corrosion resistance of the Mg matrix [140]. At the same electrolyte concentration and current density, the deposition temperature affected the activity of ions in the solution and thus became a decisive factor that affected the nucleation of calcium phosphate on the cathode surface. As a consequence, temperature played a major role in the coating structure; in particular, the deposited layer was constituted of substituted DCPD when it worked at lower temperature, and then it was constituted of a combination of DCPD and partial HA (substituted by Sr), and finally, when the temperature reached 85 °C, a Ca-deficient Sr-containing HA was formed. It can be seen that the diffraction peaks of the DCPD and HA phases that formed at different temperatures all shifted to the direction of lower 2θ degrees, confirming the substitution of Sr [140]. The surface morphologies of CaPs coatings obtained via electrodeposition at different temperatures reflected the different phase compositions. Interestingly, when the temperature increased, the structure of the coatings changed from horizontal scales to a vertical fibrous structure to an interwoven networked structure. The behavior of the (002) diffraction peak indicated that the growth direction of the crystals was perpendicular to the electrode surface during the electrodeposition, which corresponded to morphological observation [140].

**Figure 10:**
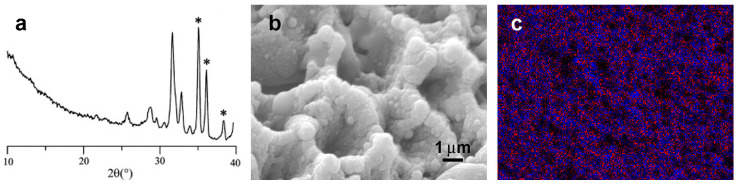
PLD coating deposited from Sr-substituted HA (Sr: 7at%) on Ti substrate: (**a**) thin film XRD pattern, the reflections due to Ti are indicated with *; (**b**) SEM micrograph; (**c**) EDS map—blue: Ca; red: Sr. Reprinted with permission from reference [141]; Copyright 2008 from Acta Materialia Inc., Elsevier.

Pulsed-laser deposition (PLD) has also proved to be a successful technique for growing thin calcium phosphate structures on metallic substrates. Coatings from targets of strontium-substituted hydroxyapatite powders (up to 7% Sr content) or from targets obtained after mixing and compacting commercial HA and SrCO_3_ powders in different proportions (up to 7% Sr content) were studied [141,142]. The resolution of the XRD patterns was always quite good (Figure 10a), indicating a relatively high degree of crystallinity of the apatitic coatings and the absence of secondary phases. Sr incorporation was confirmed via EDS and XPS. The fact that this phenomenon is linear, with a homogeneous strontium distribution on the surface of the thin films (Figure 10b), and accompanied by an equally linear withdrawal of Ca^2+^ ions of similar slope suggests that there is a substitution mechanism in the HA structure of Sr^2+^ in place of Ca^2+^. The morphology of the films was always that of a typical HA, formed by globular aggregates of a homogeneous size, with nanometer-scale roughness (Figure 10c) [141,142]. The presence of strontium in the coating enhanced osteoblast activity and differentiation, while it inhibited osteoclast production and proliferation in a dose dependent manner [141].

Matrix-Assisted Pulsed Laser Evaporation (MAPLE) was first introduced as an alternative to PLD for the synthesis of organic coatings but proved to be beneficial for the fabrication of inorganic and hybrid organic/inorganic layers as well. Using this technology, it was possible to deposit Sr-substituted octacalcium phosphate that preserved the structural role of the water molecules. The coatings, which were composed of crystal fragments, cauliflower-like aggregates, and droplets, exhibited a higher crystallinity than OCP films obtained via PLD and displayed a homogeneous distribution of strontium on the surface [143]. The MAPLE technique was also successfully applied in the combinatorial mode (C-MAPLE) to deposit gradient thin films with variable compositions of Sr-substituted HA, together with other functionalized calcium phosphates, on titanium substrates [144,145]. The surfaces of all layers were characterized by a granular morphology, with grain dimensions in the order of tens of nanometers, which did not exhibit significant variation as a function of composition, even if EDS analysis and maps confirmed the different contents of Sr and its homogeneous distribution in the different samples. The presence of Sr in the coatings always promoted osteoblast proliferation and differentiation, regardless of the crystalline phase deposited by MAPLE [143,144,145]. However, in all MAPLE studies, no clear evidence of ion substitution or peak shifts in XRD patterns was shown, despite the good crystallinity and consequent sharpness of the peaks.

## 5. Concluding Remarks

The components of the family of calcium orthophosphates display a number of similarities. However, they exhibit different structures, morphologies, and stabilities in solutions, as well as different reactions to heat treatment. Thus, it is not surprising that substituting calcium with strontium occurs to very different extents: from just 10 at% in α-TCP up to 100 at% in HA and DCPA. The presence of strontium in the structure of β-TCP improves its thermal stability, whereas the thermal stability of OCP and DCPD decreases with increasing Sr substitution. Furthermore, strontium delays the hydrolysis reaction of α-TCP in solutions and influences that of DCPD in a dose-dependent way. Again, the impact of strontium on the morphology of CaPs is almost insignificant in the case of α- and β-TCP, whereas it is remarkable for DCPD and DCPA.

The important biological role of Sr ions, which promote bone formation and inhibit abnormal bone resorption, is well documented. However, the number of studies on Sr-substituted CaPs as biomaterials is quite limited in comparison with those on Sr-doped CaPs. Further investigation into this topic would provide a deeper knowledge of the different effects of chemistry, structure, morphology, and ion release on the biological performance of these materials.

## Figures and Tables

**Table 1:** Calcium orthophosphates.

Formula	Name (Mineral)	Abbreviation
Ca_10_(PO_4_)_6_(OH)_2_	Hydroxyapatite	HA
Ca_8_H_2_(PO_4_)_6_·5H_2_O	Octacalcium phosphate	OCP
β-Ca_3_(PO_4_)_2_	Beta-tricalcium phosphate (whitlockite)	β-TCP
α-Ca_3_(PO_4_)_2_	Alpha-tricalcium phosphate	α-TCP
Ca(H_2_PO_4_)_2_·H_2_O	Monocalcium phosphate monohydrate	MCPM
Ca(H_2_PO4)_2_	Anhydrous monocalcium phosphate	MCPA
CaHPO_4_·2H_2_O	Dicalcium phosphate dihydrate (brushite)	DCPD
CaHPO_4_	Anhydrous dicalcium phosphate (monetite)	DCPA
Ca_4_(PO_4_)_2_	Tetracalcium phosphate	TTCP

## References

[1] Musgrove M.L. (2021). The occurrence and distribution of strontium in U.S. roundwater. Appl. Geochem..

[2] Boivin G., Deloffre P., Perrat B., Panczer G., Boudeulle M., Mauras Y., Allain P., Tsouderos Y., Meunier P.J. (2009). Strontium distribution and interactions with bone mineral in monkey iliac bone after strontium salt (S 12911) administration. J. Bone Miner. Res..

[3] Dahl S.G., Allain P., Marie P., Mauras Y., Boivin G., Ammann P., Tsouderos Y., Delmas P., Christiansen C. (2001). Incorporation and distribution of strontium in bone. Bone.

[4] Marx D., Rahimnejad Yazdi A., Papini M., Towler M. (2020). A review of the latest insights into the mechanism of action of strontium in bone. Bone Rep..

[5] Bussola Tovani C., Divoux T., Manneville S., Azaïs T., Laurent G., de Frutos M., Gloter A., Ciancaglini P., Ramos A.P., Nassif N. (2023). Strontium-driven physiological to pathological transition of bone-like architecture: A dose-dependent investigation. Acta Biomater..

[6] Uskokovic V., Jankovic-Castvan I., Wu V.M. (2019). Bone mineral crystallinity governs the orchestration of ossification and resorption during bone remodeling. ACS Biomater. Sci. Eng..

[7] Zhuang Y., Liu A., Jiang U., Liaqat K., Lin K., Sun W., Yuan C. (2023). Promoting vascularized bone regeneration via strontium-incorporated hydroxyapatite bioceramic. Mater. Des..

[8] Saidak Z., Marie P.J. (2012). Strontium signaling: Molecular mechanisms and therapeutic implications in osteoporosis. Pharmacol. Ther..

[9] Marie P.J., Hott M., Modrowski D., De Pollak C., Guillemain J., Deloffre P., Tsouderos Y. (1993). An uncoupling agent containing strontium prevents bone loss by depressing bone resorption and maintaining bone formation in estrogen-deficient rats. J. Bone Miner. Res..

[10] Chou J., Valenzuela S.M., Santos J., Bishop D., Milthorpe B., Green D.W., Otsuka M., Ben-Nissan B. (2014). Strontium- and magnesium-enriched biomimetic β-TCP macrospheres with potential for bone tissue morphogenesis. J. Tissue Eng. Regen. Med..

[11] Pilmane M., Salma-Ancane K., Loca D., Locs J., Berzina-Cimdina L. (2017). Strontium and strontium ranelate: Historical review of some of their functions. Mater. Sci. Eng. C-Mater. Biol. Appl..

[12] Borciani G., Ciapetti C., Vitale-Brovarone N., Baldini N. (2022). Strontium functionalization of biomaterials for bone tissue engineering purposes: A biological point of view. Materials.

[13] Kołodziejska B., Stępień N., Kolmas J. (2021). The influence of strontium on bone tissue metabolism and its application in osteoporosis treatment. Int. J. Mol. Sci..

[14] Christensen T.E., Berglund Davidsen M., Van Malderen S., Garrevoet J., Offermanns V., Andersen O.Z., Foss M., Birkedal H. (2022). Local release of strontium from sputter-deposited coatings at implants increases the strontium-to-calcium ratio in peri-implant bone. ACS Biomater. Sci. Eng..

[15] Sheng X., Li C., Wang Z., Xu Y., Sun Y., Zhang W., Liu H., Wang J. (2023). Advanced applications of strontium-containing biomaterials in bone tissue engineering. Materials Today Bio.

[16] Bigi A., Boanini E., Gazzano M., Aparicio C., Ginebra M.P. (2015). Ion substitution in biological and synthetic apatites. Biomineralization and Biomaterials, Foundamentals and Applications.

[17] Shah F.A. (2025). Revisiting the physical and chemical nature of the mineral component of bone. Acta Biomater..

[18] Boanini E., Gazzano M., Bigi A. (2010). Ionic substitutions in calcium phosphates synthesized at low temperature. Acta Biomater..

[19] LeGeros Z.R., Ito A., Ishikawa K., Sakae T., LeGeros J.P., Basu B., Katti D.S., Kumar A. (2009). Fundamentals of hydroxyapatite and related calcium phosphates. Advanced Biomaterials: Fundamentals, Processing, and Applications.

[20] Bigi A., Boanini E. (2017). Functionalized biomimetic calcium phosphates for bone tissue repair. J. Appl. Biomater. Funct. Mater..

[21] Elliott J.C. (1994). Structure and Chemistry of the Apatites and Other Calcium Orthophosphates.

[22] Dorozhkin S.V., Epple M. (2002). Biological and medical significance of calcium phosphates. Angew. Chem. Int. Ed..

[23] Durucan C., Brown P.W. (2002). Reactivity of alpha-tricalcium phosphate. J. Mater. Sci..

[24] Boanini E., Panzavolta S., Rubini K., Gandolfi M., Bigi A. (2010). Effect of strontium and gelatin on the reactivity of α-tricalcium phosphate. Acta Biomater..

[25] Zhang J., Wang L., Putnis C.V. (2019). Underlying role of brushite in pathological mineralization of hydroxyapatite. J. Phys. Chem. B.

[26] Bigi A., Boanini E., Suzuki O., Insley G. (2020). Functionalization of octacalcium phosphate for bone replacement. Octacalcium Phosphate Biomaterials Understanding of Bioactive Properties and Application.

[27] Suzuki O., Hamai R., Sakai S. (2023). The material design of octacalcium phosphate bone substitute: Increased dissolution and osteogenecity. Acta Biomater..

[28] Tung M.S., Tomazic B., Brown W.E. (1992). The effects of magnesium and fluoride on the hydrolysis of octacalcium phosphate. Arch. Oral Biol..

[29] Chow L.C., Markovic M., Frukhtbeyn S.A., Takagi S. (2005). Hydrolysis of tetracalcium phosphate under a near-constant-composition condition—Effects of pH and particle size. Biomaterials.

[30] Šupová M. (2015). Substituted hydroxyapatites for biomedical applications: A review. Ceram. Int..

[31] Ressler A., Žužić A., Ivanišević I., Kamboj N., Ivanković H. (2021). Ionic substituted hydroxyapatite for bone regeneration applications: A review. Open Ceram..

[32] Bigi A., Boanini E., Capuccini C., Gazzano M. (2007). Strontium-substituted hydroxyapatite nanocrystals. Inorg. Chim. Acta.

[33] Zhu K., Yanagisawa K., Shimanouchi R., Onda A., Kajiyoshi K. (2006). Preferential occupancy of metal ions in the hydroxyapatite solid solutions synthesized by hydrothermal method. J. Eur. Ceram. Soc..

[34] O’Donnell M.D., Fredholm Y., de Rouffignac A., Hill R.G. (2008). Structural analysis of a series of strontium-substituted apatites. Acta Biomater..

[35] Zeglinski J., Nolan M., Bredol M., Schattec A., Tofail S.A.M. (2012). Unravelling the specific site preference in doping of calcium hydroxyapatite with strontium from ab initio investigations and Rietveld analyses. Phys. Chem. Chem. Phys..

[36] Terra J., Dourado E.R., Eon J.G., Ellis D.E., Gonzalez G., Rossi A.M. (2009). The structure of strontium-doped hydroxyapatite: An experimental and theoretical study. Phys. Chem. Chem. Phys..

[37] Kikuchi M., Yamazaki A., Otsuka R., Akao M., Aoki H. (1994). Crystal structure of Sr-substituted hydroxyapatite synthesized by hydrothermal method. J. Solid State Chem..

[38] Baldassarre F., Altomare A., Mesto E., Lacalamita M., Dida B., Mele A., Bauer E.M., Puzone M., Tempesta E., Capelli D. (2023). Structural characterization of low-Sr-doped hydroxyapatite obtained by solid-state synthesis. Crystals.

[39] Matsunaga K., Murata H. (2009). Strontium substitution in bioactive calcium phosphates: A first-principles study. J. Phys. Chem. B.

[40] Wang M., Wang Y., Tian Y., Zhu Y. (2022). Anisotropic expansion effect of Sr doping on the crystal structure of hydroxyapatite. CrystEngComm.

[41] Pan H.B., Li Z.Y., Lam W.M., Wong J.C., Darvell B.W., Luk K.D., Lu W.W. (2009). Solubility of strontium-substituted apatite by solid titration. Acta Biomater..

[42] Cheng G., Zhang Y., Yin H., Ruan Y., Sun Y., Lin K. (2019). Effects of strontium substitution on the structural distortion of hydroxyapatite by Rietveld refinement and Raman spectroscopy. Ceram. Int..

[43] Shi H., He F., Ye J. (2016). Synthesis and structure of iron- and strontium-substituted octacalcium phosphate: Effects of ionic charge and radius. J. Mater. Chem. B.

[44] Boanini E., Gazzano M., Rubini K., Bigi A. (2010). Collapsed octacalcium phosphate stabilized by ionic substitutions. Cryst. Growth Des..

[45] Teterina A.Y., Smirnov I.V., Fadeeva I.S., Fadeev R.S., Smirnova P.V., Minaychev V.V., Kobyakova M.I., Fedotov A.Y., Barinov S.M., Komlev V.S. (2021). Octacalcium phosphate for bone tissue engineering: Synthesis, modification, and in vitro biocompatibility assessment. Int. J. Mol. Sci..

[46] Shi H., Ye X., Wu T., Zhang J., Ye J. (2017). Regulating the physicochemical and biological properties in vitro of octacalcium phosphate by substitution with strontium in a large doping range. Mater. Today Chem..

[47] Ressler A., Cvetnic M., Antunovic M., Marijanovic I., Ivankovic M., Ivankovic H. (2020). Strontium substituted biomimetic calcium phosphate system derived from cuttlefish bone. J. Biomed. Mater. Res. B Appl. Biomater..

[48] Shi H., Ye X., Zhang J., Wu T., Yu T., Zhou C., Ye J. (2021). A thermostability perspective on enhancing physicochemical and cytological characteristics of octacalcium phosphate by doping iron and strontium. Bioact. Mater..

[49] Yashima M., Sakai A., Kamiyama T., Hoshikawa A. (2003). Crystal structure analysis of β-tricalcium phosphate Ca_3_(PO_4_)_2_ by neutron powder diffraction. J. Solid State Chem..

[50] Kannan S., Pina S., Ferreira J.M.F. (2006). Formation of strontium-stabilized β-tricalcium phosphate from calcium-deficient apatite. J. Am. Ceram. Soc..

[51] Renaudin G., Laquerriere P., Filinchuk Y., Jallot E., Nedelec J.M. (2008). Structural characterization of sol–gel derived Sr-substituted calcium phosphates with anti-osteoporotic and anti-inflammatory properties. J. Mater. Chem..

[52] Belik A.A., Izumi F., Stefanovich S.Y., Malakho A.P., Lazoryak B.I., Leonidov I.A., Leonidova O.N., Davydov S.A. (2002). Polar and centrosymmetric phases in solid solutions Ca_3−x_Sr_x_(PO_4_)_2_ (0 ≤ x ≤ 16/7). Chem. Mater..

[53] Naciri Y., Hsini A., Ajmal Z., Bouddouch A., Bakiz B., Navío J.A., Albourine A., Valmalette J.-C., Ezahri M., Benlhachemi A. (2020). Influence of Sr-doping on structural, optical and photocatalytic properties of synthesized Ca_3_(PO_4_)_2_. J. Colloid Interf. Sci..

[54] Bigi A., Foresti E., Gandolfi M., Gazzano M., Roveri N. (1997). Isomorphous substitutions in β-tricalcium phosphate: The different effects of zinc and strontium. J. Inorg. Biochem..

[55] Boanini E., Gazzano M., Nervi C., Chierotti M.R., Rubini K., Gobetto R., Bigi A. (2019). Strontium and zinc substitution in β-tricalcium phosphate: An X-ray diffraction, solid state NMR and ATR-FTIR study. J. Funct. Biomater..

[56] Fadeeva I.V., Deyneko D.V., Forysenkova A.A., Morozov V.A., Akhmedova S.A., Kirsanova V.A., Sviridova I.K., Sergeeva N.S., Rodionov S.A., Udyanskaya I.L. (2022). Strontium substituted β-tricalcium phosphate ceramics: Physiochemical properties and cytocompatibility. Molecules.

[57] Somers N., Jean F., Lasgorceix M., Curto H., Urruth G., Thuault A., Petit F., Leriche A. (2021). Influence of dopants on thermal stability and densification of β-tricalcium phosphate powders. Open Ceram..

[58] Saint-Jean S.J., Camiré C.L., Nevsten P., Hansen S., Ginebra M.P. (2005). Study of the reactivity and in vitro bioactivity of Sr-substituted α-TCP cements. J. Mater. Sci.-Mater. Med..

[59] Gazzano M., Rubini K., Bigi A., Boanini E. (2023). Strontium-substituted α-TCP: Structure, stability, and reactivity in solution. Cryst. Growth Des..

[60] Matsunaga K., Kubota T., Toyoura K., Nakamura A. (2015). First-principles calculations of divalent substitution of Ca^2+^ in tricalcium phosphates. Acta Biomater..

[61] Schumacher M., Gelinsky M. (2015). Strontium modified calcium phosphate cements—Approaches towards targeted stimulation of bone turnover. J. Mater. Chem. B.

[62] Lodoso-Torrecilla I., van den Beucken J.J.J.P., Jansen J.A. (2021). Calcium phosphate cements: Optimization toward biodegradability. Acta Biomater..

[63] Sayahi M., Santos J., El-Feki H., Charvillat C., Bosc F., Karacan I., Milthorpe B., Drouet C. (2020). Brushite (Ca,M)HPO_4_ 2H_2_O doping with bioactive ions (M = Mg^2+^, Sr^2+^, Zn^2+^, Cu^2+^, and Ag^+^): A new path to functional biomaterials?. Mater. Today Chem..

[64] Boanini E., Silingardi F., Gazzano M., Bigi A. (2021). Synthesis and hydrolysis of brushite (DCPD): The role of ionic substitution. Cryst. Growth Des..

[65] Fowler B.O. (1974). Infrared studies of apatites. II. Preparation of normal and isotopically substituted calcium, strontium, and barium hydroxyapatites and spectra-structure-composition correlations. Inorg. Chem..

[66] Boanini E., Gazzano M., Rubini K., Mazzeo P.P., Bigi A. (2021). Structural interplay between strontium and calcium in α-CaHPO4 and β-SrHPO4. Ceram. Int..

[67] Catti M., Ferraris G., Filhol A. (1977). Hydrogen bonding in the crystalline state. CaHPO_4_ (Monetite), P-1 or PI? A novel neutron diffraction study. Acta Crystallogr. B.

[68] Jayasree R., Kumar T.S.S., Mahalaxmi S., Abburi S., Rubaiya Y., Doble M. (2017). Dentin remineralizing ability and enhanced antibacterial activity of strontium and hydroxyl ion co-releasing radiopaque hydroxyapatite cement. J. Mater. Sci. Mater. Med..

[69] Delgado López J.M., Frison R., Cervellino A., Gómez-Morales J., Guagliardi A., Masciocchi N. (2014). Crystal size, morphology, and growth mechanism in bio-inspired apatite nanocrystals. Adv. Funct. Mater..

[70] Geng Z., Cui Z., Li Z., Zhu S., Liang Y., Lu W.W., Yang X. (2015). Synthesis, characterization and the formation mechanism of magnesium- and strontium-substituted hydroxyapatite. J. Mater. Chem. B.

[71] Kurzyk A., Szwed-Georgiou A., Pagacz J., Antosik A., Tymowicz-Grzyb P., Gerle A., Szterner P., Włodarczyk M., Płociński P., Urbaniak M.M. (2023). Calcination and ion substitution improve physicochemical and biological properties of nanohydroxyapatite for bone tissue engineering applications. Sci. Rep..

[72] Bigi A., Boanini E., Gazzano M., Rubini K. (2005). Structural and morphological modifications of hydroxyapatite-polyaspartate composite crystals induced by heat treatment. Cryst. Res. Technol..

[73] Jokić B., Mitrić M., Radmilović V., Drmanić S., Petrović R., Janaćković D. (2011). Synthesis and characterization of monetite and hydroxyapatite whiskers obtained by a hydrothermal method. Ceram. Int..

[74] Stojanović Z.S., Ignjatović N., Wu V., Žunič V., Veselinović L., Škapin S., Miljković M., Uskoković V., Uskoković D. (2016). Hydrothermally processed 1D hydroxyapatite: Mechanism of formation and biocompatibility studies. Mater. Sci. Eng. C-Mater. Biol. Appl..

[75] Méndez-Lozano N., Velázquez-Castillo R., Rivera-Muñoz E.M., Bucio-Galindo L., Mondragón-Galicia G., Manzano-Ramírez A., Ángel Ocampo M., Apátiga-Castro L.M. (2017). Crystal growth and structural analysis of hydroxyapatite nanofibers synthesized by the hydrothermal microwave-assisted method. Ceram. Int..

[76] Ren X., Liang Z., Zhao X. (2023). Preparation of hydroxyapatite nanofibers by using ionic liquids as template and application in enhancing hydrogel performance. Front. Bioeng. Biotechnol..

[77] Ye Z., Qi Y., Zhang A., Karels B.J., Aparicio C. (2023). Biomimetic mineralization of fibrillar collagen with strontium-doped hydroxyapatite. ACS Macro Lett..

[78] Bigi A., Boanini E., Falini G., Panzavolta S., Roveri N. (2000). Effect of sodium polyacrylate on the hydrolysis of octacalcium phosphate. J. Inorg. Biochem..

[79] Sinusaite L., Grigoraviciute-Puroniene I., Popov A., Ishikawa K., Kareiva A., Zarkov A. (2019). Controllable synthesis of tricalcium phosphate (TCP) polymorphs by wet precipitation: Effect of washing procedure. Ceram. Int..

[80] Ruiz-Aguilar C., Olivares-Pinto U., Aguilar-Reyes E.A., López-Juárez R., Alfonso I. (2018). Characterization of β-tricalcium phosphate powders synthesized by sol–gel and mechanosynthesis. Bol. Soc. Esp. Ceram. Vidr..

[81] Yuan Z., Bi J., Wang W., Sun X., Wang L., Mao J., Yang F. (2021). Synthesis and properties of Sr2+ doping α-tricalcium phosphate at low temperature. J. Appl. Biomater. Funct. Mater..

[82] Švecová M., Bartůněk V. (2018). Facile synthesis of monetite nanoparticles from basic raw materials. Ceram. Int..

[83] Chen S., Krumova M., Cölfen H., Sturm E.V. (2019). Synthesis of fiber-like monetite without organic additives and its transformation to hydroxyapatite. Chem. Mater..

[84] Adawy A., Diaz R. (2022). Probing the structure, cytocompatibility, and antimicrobial efficacy of silver-, strontium-, and zinc-doped monetite. ACS Appl. Bio Mater..

[85] Rabelo Neto J.S., Ricardo P.C., Valério M.E.G., Xia W., Engqvist H., Fredel M.C. (2024). The influence of strontium doping on the crystal morphology of synthetic calcium phosphates. J. Mol. Struct..

[86] Bigi A., Boanini E. (2018). Calcium phosphates as delivery systems for bisphosphonates. J. Funct. Biomater..

[87] Zeng Y., Hoque J., Varghese S. (2019). Biomaterial-assisted local and systemic delivery of bioactive agents for bone repair. Acta Biomater..

[88] Eliaz N., Metoki N. (2017). Calcium phosphate bioceramics: A review of their history, structure, properties, coating technologies and biomedical applications. Materials.

[89] Lukaviciute L., Ganceviciene R., Tsuru K., Ishikawa K., Yang J.C., Grigoraviciute I., Kareiva A. (2024). Cationic substitution effects in phosphate-based bioceramics—A way towards superior bioproperties. Ceram. Int..

[90] Zhang J., Liu W., Schnitzler V., Tancret F., Bouler J.M. (2014). Calcium phosphate cements for bone substitution: Chemistry, handling and mechanical properties. Acta Biomater..

[91] Schumacher M., Henß A., Rohnke M., Gelinsky M. (2013). A novel and easy-to-prepare strontium(II) modified calcium phosphate bone cement with enhanced mechanical properties. Acta Biomater..

[92] Schumacher M., Lode A., Helth A., Gelinsky M. (2013). A novel strontium(II)-modified calcium phosphate bone cement stimulates human-bone-marrow-derived mesenchymal stem cell proliferation and osteogenic differentiation in vitro. Acta Biomater..

[93] Schumacher M., Wagner A.S., Kokesch-Himmelreich J., Bernhardt A., Rohnke M., Wenisch S., Gelinsky M. (2016). Strontium substitution in apatitic CaP cements effectively attenuates osteoclastic resorption but does not inhibit osteoclastogenesis. Acta Biomater..

[94] Lode A., Heiss C., Knapp G., Thomas J., Nies B., Gelinsky M., Schumacher M. (2018). Strontium-modified premixed calcium phosphate cements for the therapy of osteoporotic bone defects. Acta Biomater..

[95] Tadier S., Bareille R., Siadous R., Marsan O., Charvillat C., Cazalbou S., Amedee J., Rey C., Combes C. (2012). Strontium-loaded mineral bone cements as sustained release systems: Compositions, release properties, and effects on human osteoprogenitor cells. J. Biomed. Mater. Res. Part B.

[96] Guo D., Xu K., Zhao X., Han Y. (2005). Development of a strontium-containing hydroxyapatite bone cement. Biomaterials.

[97] Hamdan Alkhraisat M., Tamimi Mariño F., Rueda Rodríguez C., Blanco Jerez L., Lopez Cabarcos E. (2008). Combined effect of strontium and pyrophosphate on the properties of brushite cements. Acta Biomater..

[98] Hamdan Alkhraisat M., Moseke C., Blanco L., Barralet J.E., Lopez-Carbacos E., Gbureck U. (2008). Strontium modified biocements with zero order release kinetics. Biomaterials.

[99] Hamdan Alkhraisat M., Rueda C., Cabrejos-Azama J., Lucas-Aparicio J., Tamimi Mariño F., Torres García-Denche J., Blanco Jerez L., Gbureck U., Lopez Cabarcos E. (2010). Loading and release of doxycycline hyclate from strontium-substituted calcium phosphate cement. Acta Biomater..

[100] Taha A., Akram M., Jawad Z., Alshemary A.Z., Hussain R. (2017). Strontium doped injectable bone cement for potential drug delivery applications. Mater. Sci. Eng. C Mater. Biol. Appl..

[101] Rau J.V., Fadeeva I.V., Forysenkova A.A., Davydova G.A., Fosca M., Filippov Y.Y., Antoniac I.V., Antoniac A., D’Arco A., Di Fabrizio M. (2022). Strontium substituted tricalcium phosphate bone cement: Short and long-term time-resolved studies and in vitro properties. Adv. Mater. Interfaces.

[102] Hamdan Alkhraisat M., Rueda C., López Cabarcos E. (2011). Strontium ions substitution in brushite crystals: The role of strontium chloride. J. Funct. Biomater..

[103] Pina S., Torres P.M., Goetz-Neunhoeffer F., Neubauer J., Ferreira J.M.F. (2010). Newly developed Sr-substituted α-TCP bone cements. Acta Biomater..

[104] Silingardi F., Salamanna F., Español M., Maglio M., Sartori M., Giavaresi G., Bigi A., Ginebra M.P., Boanini E. (2024). Regulation of osteogenesis and angiogenesis by cobalt, manganese and strontium doped apatitic materials for functional bone tissue regeneration. Biomater. Adv..

[105] Hurle K., Oliveira J.M., Reis R.L., Pina S., Goetz-Neunhoeffer F. (2021). Ion-doped brushite cements for bone regeneration. Acta Biomater..

[106] Dai J., Fu Y., Chen D., Sun Z. (2021). A novel and injectable strontium-containing hydroxyapatite bone cement for bone substitution: A systematic evaluation. Mater. Sci. Eng. C-Mater. Biol. Appl..

[107] Neves N., Linhares D., Costa G., Ribeiro C.C., Barbosa M.A. (2017). In vivo and clinical application of strontium-enriched biomaterials for bone regeneration: A systematic review. Bone Joint Res..

[108] Thormann U., Ray S., Sommer U., ElKhassawna T., Rehling T., Hundgeburth M., Henß A., Rohnke M., Janek J., Lips K.S. (2013). Bone formation induced by strontium modified calcium phosphate cement in critical-size metaphyseal fracture defects in ovariectomized rats. Biomaterials.

[109] Roseti L., Parisi V., Petretta M., Cavallo C., Desando G., Bartolotti I., Grigolo B. (2017). Scaffolds for bone tissue engineering: State of the art and new perspectives. Mater. Sci. Eng. C-Mater. Biol. Appl..

[110] Butscher A., Bohner M., Hofmann S., Gauckler L., Müller R. (2011). Structural and material approaches to bone tissue engineering in powder-based three-dimensional printing. Acta Biomater..

[111] Panzavolta S., Fini M., Nicoletti A., Bracci B., Rubini K., Giardino R., Bigi A. (2009). Porous composite scaffolds based on gelatin and partially hydrolyzed alpha-tricalcium phosphate. Acta Biomater..

[112] Zhang L., Yang G., Johnson B.N., Jia X. (2019). Three-dimensional (3D) printed scaffold and material selection for bone repair. Acta Biomater..

[113] Pedde R.D., Mirani B., Navaei A., Styan T., Wong S., Mehrali M., Thakur A., Mohtaram N.K., Bayati A., Dolatshahi-Pirouz A. (2017). Emerging biofabrication strategies for engineering complex tissue constructs. Adv. Mater..

[114] Chen T., Luo L., Li J., Li J., Lin T., Liu M., Sang H., Hong X., Pu J., Huang W. (2025). Advancements in 3D printing technologies for personalized treatment of osteonecrosis of the femoral head. Mater. Today Bio.

[115] Panzavolta S., Torricelli P., Casolari S., Parrilli A., Fini M., Bigi A. (2018). Strontium-substituted hydroxyapatite-gelatin biomimetic scaffolds modulate bone cell response. Macromol. Biosci..

[116] Lei Y., Xu Z., Ke Q., Yin W., Chen Y., Zhang C., Guo Y. (2017). Strontium hydroxyapatite/chitosan nanohybrid scaffolds with enhanced osteoinductivity for bone tissue engineering. Mater. Sci. Eng. C Mater. Biol. Appl..

[117] Ehret C., Aid-Launais R., Sagardoy T., Siadous R., Bareille R., Rey S., Pechev S., Etienne L., Kalisky J., de Mones E. (2017). Strontium-doped hydroxyapatite polysaccharide materials effect on ectopic bone formation. PLoS ONE.

[118] Quade M., Schumacher M., Bernhardt A., Lode A., Kampschulte M., Voß A., Simon P., Uckermann O., Kirsch M., Gelinsky M. (2018). Strontium-modification of porous scaffolds from mineralized collagen for potential use in bone defect therapy. Mater. Sci. Eng. C Mater. Biol. Appl..

[119] Wu Y.C., Lin W.Y., Yang C.Y., Lee T.M. (2015). Fabrication of gelatin-strontium substituted calcium phosphate scaffolds with unidirectional pores for bone tissue engineering. J. Mater. Sci. Mater. Med..

[120] Shaikh S., Mehrotra S., van Bochove B., Teotia A.K., Singh P., Laurén I., Lindfors N.C., Seppälä J., Kumar A. (2024). Strontium-substituted nanohydroxyapatite containing biodegradable 3D printed composite scaffolds for bone regeneration. ACS Appl. Mater. Interfaces.

[121] Liu D., Nie W., Li D., Wang W., Zheng L., Zhang J., Zhang J., Peng C., Mo X., He C. (2019). 3D printed PCL/SrHA scaffold for enhanced bone regeneration. Chem. Eng. J..

[122] Fischetti T., Borciani G., Avnet S., Rubini K., Baldini N., Graziani G., Boanini E. (2023). Incorporation/enrichment of 3D bioprinted constructs by biomimetic nanoparticles: Tuning printability and cell behavior in bone models. Nanomaterials.

[123] Huang W., Li Z., Xiong J., Zhang C., Gan J., Fu Q., Li Y., Wen R., He F., Shi H. (2024). Fabrication of 3D-printed Ca_2_Sr(PO_4_)_2_-based composite ceramic scaffolds as potential bone regenerative biomaterials. Ceram. Int..

[124] M’Pemba Hennebert P., Amirthalingam S., Hoon Kang T., So K.H., Hwang N.S. (2024). Strontium-doped whitlockite scaffolds for enhanced bone regeneration. ACS Appl. Mater. Interfaces.

[125] Zhao R., Chen S., Zhao W., Yang L., Yuan B., Ioan V.S., Iulian A.V., Yang X., Zhu X., Zhang X. (2020). A bioceramic scaffold composed of strontium-doped three-dimensional hydroxyapatite whiskers for enhanced bone regeneration in osteoporotic defects. Theranostics.

[126] Tao Z.S., Zhou W.S., He X.W., Liu W., Bai B.L., Zhou Q., Huang Z.L., Tu K.K., Li H., Sun T. (2016). A comparative study of zinc, magnesium, strontium-incorporated hydroxyapatite-coated titanium implants for osseointegration of osteopenic rats. Mater. Sci. Eng. C Mater. Biol. Appl..

[127] Xue W., Hosick H.L., Bandyopadhyay A., Bose S., Ding C., Luk K.D.K., Cheung K.M.C., Lu W.W. (2007). Preparation and cell–materials interactions of plasma sprayed strontium-containing hydroxyapatite coating. Surf. Coat. Technol..

[128] Ullah I., Xu Q., Ullah Jan H., Ren L., Yang K. (2022). Effects of strontium and zinc substituted plasma sprayed hydroxyapatite coating on bone-like apatite layer formation and cell-material interaction. Mat. Chem. Phys..

[129] Fielding G.A., Roy M., Bandyopadhyay A., Bose S. (2012). Antibacterial and biological characteristics of silver containing and strontium doped plasma sprayed hydroxyapatite coatings. Acta Biomater..

[130] Bansal P., Singh G., Sidhu H.S. (2021). Plasma-sprayed hydroxyapatite-strontium coating for improved corrosion resistance and surface properties of biodegradable AZ31 Mg alloy for biomedical applications. J. Mater. Eng. Perform..

[131] Ghezzi D., Graziani G., Cappelletti M., Fadeeva I.V., Montesissa M., Sassoni E., Borciani G., Barbaro K., Boi M., Baldini N. (2024). New strontium-based coatings show activity against pathogenic bacteria in spine infection. Front. Bioeng. Biotechnol..

[132] Oliveira A.L., Reis R.L., Li P. (2007). Strontium-substituted apatite coating grown on Ti6Al4V substrate through biomimetic synthesis. J. Biomed. Mater. Res. B Appl. Biomater..

[133] Bracci B., Torricelli P., Panzavolta S., Boanini E., Giardino R., Bigi A. (2009). Effect of Mg^2+^, Sr^2+^, and Mn^2+^ on the chemico-physical and in vitro biological properties of calcium phosphate biomimetic coatings. J. Inorg. Biochem..

[134] Xia W., Lindahl C., Lausmaa J., Borchardt P., Ballo A., Thomsen P., Engqvist H. (2010). Biomineralized strontium-substituted apatite/titanium dioxide coating on titanium surfaces. Acta Biomater..

[135] Navarro da Rocha D., de Oliveira Cruz L.R., de Campos J.B., Lopes dos Santos J., Santana Blazutti Marçal R.L., Mijares D.Q., Maza Barbosa R., Coelho P.G., Prado da Silva M.H. (2019). Bioactivity of strontium-monetite coatings for biomedical applications. Ceram. Int..

[136] Wen J., Gu G.C., Wang K., Xiao G.Y., Lu Y.P., Liu B. (2024). Synthesis and characterization of strontium substituted monetite coatings via mild phosphate chemical conversion. Surf. Coat. Technol..

[137] Ozeki K., Goto T., Aoki H., Masuzawa T. (2014). Characterization of Sr-substituted hydroxyapatite thin film by sputtering technique from mixture targets of hydroxyapatite and strontium apatite. Biomed. Mater. Eng..

[138] Boyd A.R., Rutledge L., Randolph L.D., Meenan B.J. (2015). Strontium-substituted hydroxyapatite coatings deposited via a co-deposition sputter technique. Mater. Sci. Eng. C Mater. Biol. Appl..

[139] Drevet R., Benhayoune H. (2013). Pulsed electrodeposition for the synthesis of strontium-substituted calcium phosphate coatings with improved dissolution properties. Mater. Sci. Eng. C Mater. Biol. Appl..

[140] Xu Y., Li G., Zhang Z., Lian J., Guo Y., Ren L. (2024). Effect of strontium-substituted calcium phosphate coatings prepared by one-step electrodeposition at different temperatures on corrosion resistance and biocompatibility of AZ31 magnesium alloys. ACS Biomater. Sci. Eng..

[141] Capuccini C., Torricelli P., Sima F., Boanini E., Ristoscu C., Bracci B., Socol G., Fini M., Mihailescu I.N., Bigi A. (2008). Strontium-substituted hydroxyapatite coatings synthesized by pulsed-laser deposition: In vitro osteoblast and osteoclast response. Acta Biomater..

[142] Pereiro I., Rodríguez-Valencia C., Serra C., Solla E.L., Serra J., González P. (2012). Pulsed laser deposition of strontium-substituted hydroxyapatite coatings. Appl. Surf. Sci..

[143] Boanini E., Torricelli P., Fini M., Sima F., Serban N., Mihailescu I.N., Bigi A. (2012). Magnesium and strontium doped octacalcium phosphate thin films by matrix assisted pulsed laser evaporation. J. Inorg. Biochem..

[144] Boanini E., Torricelli P., Sima F., Axente E., Fini M., Mihailescu I.N., Bigi A. (2015). Strontium and zoledronate hydroxyapatites graded composite coatings for bone prostheses. J. Colloid Interface Sci..

[145] Boanini E., Torricelli P., Sima F., Axente E., Fini M., Mihailescu I.N., Bigi A. (2018). Gradient coatings of strontium hydroxyapatite/zinc β-tricalcium phosphate as a tool to modulate osteoblast/osteoclast response. J. Inorg. Biochem..

